# Effectiveness of distal versus proximal greater occipital nerve pulsed radiofrequency in migraine management: a prospective randomized controlled trial

**DOI:** 10.55730/1300-0144.6007

**Published:** 2025-05-07

**Authors:** Gülçin BABAOĞLU, Şükriye DADALI, Ülkü SABUNCU, Erkan Yavuz AKÇABOY, Şeref ÇELİK, Mustafa Yemliha AYHAN, Yağmur Can DADAKÇI, Mustafa Cem YILMAZ, Şaziye ŞAHİN

**Affiliations:** Department of Pain Medicine, Ankara Bilkent City Hospital, Ankara, Turkiye

**Keywords:** Migraine, pulsed radiofrequency, greater occipital nerve, proximal approach, distal approach

## Abstract

**Background/aim:**

We aimed to evaluate the effectiveness of distal versus proximal greater occipital nerve (GON) pulsed radiofrequency (PRF) treatments in patients with episodic or chronic migraine.

**Materials and methods:**

In this prospective, randomized controlled study, sixty participants were randomized to either distal GON PRF (n = 30) or proximal GON PRF (n = 30). Migraine related assessments were conducted at the baseline and at the first, second, and third month.

**Results:**

Baseline characteristics indicated a higher migraine burden in the proximal group, including increased monthly headache frequency (15.0 vs. 9.5 attacks, p = 0.007). Both groups experienced significant reductions in headache duration, severity and frequency over three months (Friedman test, p < 0.001). Notably, the proximal group experienced greater reductions in severe headache frequency at all time points (1st month: p = 0.004; 3rd month: p = 0.022) and total headache days by the third month (14.0 vs. 9.5 days, p = 0.039). The distal group exhibited some advantages in reducing headache severity (VAS), showing a trend toward improvement in the second month (p = 0.055) and achieving statistical significance by the third month (p = 0.011). No unexpected adverse effects were observed in either group.

**Conclusion:**

Both treatments were well-tolerated, with minimal adverse effects. Our findings indicate that both proximal and distal approaches are safe and effective for migraine management. The proximal approach might offer slightly superior outcomes for patients experiencing severe and frequent migraine attacks.

## 1. Introduction

Migraine is a chronic and often lifelong neurological disorder that affects over one billion people with a disproportionate impact on women [1]. Although not a life-threatening disease, it is the second leading cause of disability worldwide [2].

Migraine can be categorized as either episodic or chronic types based on the frequency of headaches. Chronic migraine is characterized by headaches occurring on 15 or more days per month, with at least eight of these days being associated with migraine-like symptoms, such as pulsating pain, nausea, photophobia, and phonophobia [3]. It has been reported that 2.5% to 3% of patients with episodic migraine transform into chronic migraine within 1 year [4]. The burden of chronic migraine extends beyond the physical symptoms, significantly impairing the quality of life and often leading to economic losses due to decreased productivity and increased healthcare costs [5].

Current treatments for migraine include both pharmacological and nonpharmacological approaches [6]. Pharmacological treatments, such as prophylactic medications, NSAIDs, and triptans, are often the first line treatments. However, many patients experience inadequate relief or adverse effects from these medications, highlighting the need for alternative treatment options [4]. In recent years, nerve block techniques, particularly targeting the greater occipital nerve (GON), have gained attention as a promising nonpharmacological intervention for migraine management [7].

The greater occipital nerve, which originates from the second cervical spinal nerve (C2), is a major sensory nerve supplying the scalp. The GON is believed to play a key role in migraine pathophysiology due to the convergence between the trigeminal and upper cervical sensory neurons in the trigeminal nucleus caudalis [8,9]. GON block, which involves the injection of local anesthetic near the nerve, inhibits trigeminocervical complex and reduces headache frequency, severity, and duration in patients with migraines [10,11]. Pulsed radiofrequency (PRF) therapy is used to prolong this observed therapeutic benefit. PRF is an application that reduces pain signals through the electrical field it creates in the targeted nerves without damaging the neuronal and surrounding structures [12]. It has been reported that GON PRF can effectively manage various headaches, including migraine, occipital neuralgia, and cervicogenic headache [13]. Although the exact mechanism of action is not known, it is believed to cause neuromodulation by reducing transmission in A delta and C fibers [14].

Two different techniques have been described for GON interventions. These are the proximal technique targeting the greater occipital nerve at the level of the second cervical vertebra (C2) and the distal technique targeting the superior nuchal line level. When the proximal technique was first described by Greher et al, it was suggested that it would provide a more precise blockade compared to the distal technique [15]. However, there is a lack of consensus on the optimal site for GON interventions. Distal and proximal approaches to GON blocks have been analyzed, with varying reports on their efficacy [16–18]. There are studies comparing the efficacy of distal and proximal GON blocks, but the data is limited.

We aimed to compare the efficacy of distal and proximal GON PRF in patients with episodic or chronic migraine by evaluating changes in headache duration, severity, frequency, and the number of days with headache to provide evidence for guiding clinical decision making.

## 2. Materials and methods

### 2.1. Study population

This study was designed as a prospective randomized controlled trial. The study population consisted of patients diagnosed with episodic or chronic migraine in accordance with the International Classification of Headache Disorders (ICHD-3) criteria, recruited from a tertiary care hospital. This study included patients aged 18 to 65 years who experienced at least one migraine attack per week or four migraine attacks per month and had documented nonresponse to at least two prophylactic migraine treatments. Individuals were excluded if they had primary headache disorders other than migraines, had received preventive treatment within the past month, or had a recent history of onabotulinumtoxin A (BoNT-A) use. Additional exclusion criteria included prior nonpharmacological treatments such as acupuncture, ozone therapy, or cognitive behavioral therapy within the last six months, as well as infections at the injection site. Pregnant or breastfeeding individuals, those with a history of local anesthetic allergy, and those with a history of cranial or cervical surgery were also excluded. Furthermore, patients with conditions that could cause or worsen headaches, including coagulopathy, malignancy, uncontrolled hypertension, or intracranial lesions, were not included. Finally, individuals with psychiatric disorders or dementia that could impair treatment adherence were also excluded from the study. Patients who declined participation were also excluded.

Participants were informed of the study’s purpose, procedures, and potential risks. They were assured that their participation was voluntary and that they could withdraw from the study at any time without consequence. Data confidentiality was strictly maintained throughout the study.

**Figure 1:**
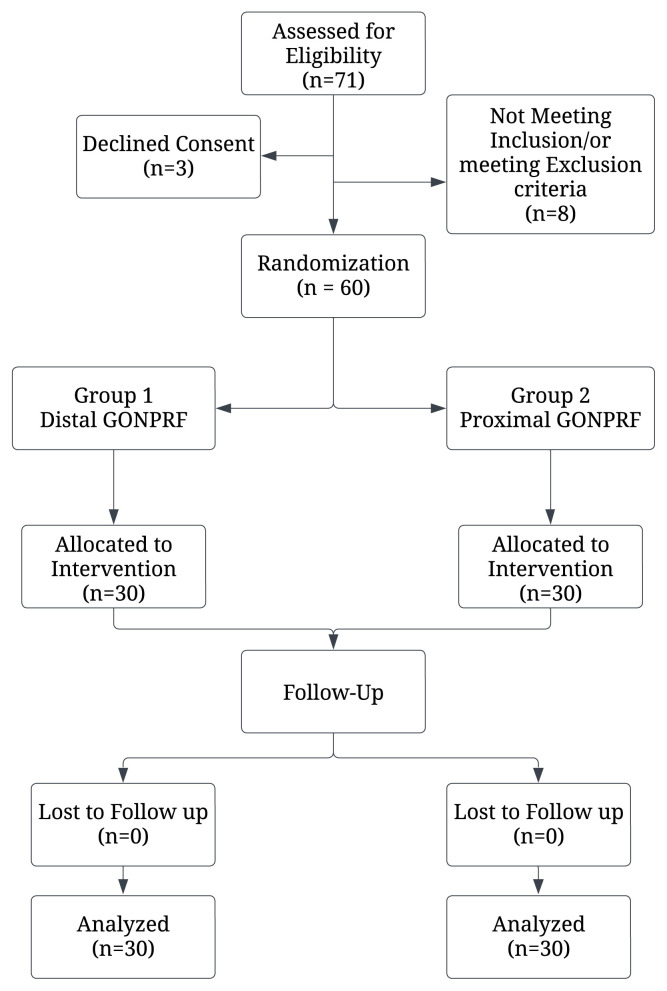
Flowchart of patient screening, randomization, group allocation, intervention, and analysis.

Following the screening of 71 patients, 60 met the eligibility criteria and were enrolled in the study. Using a basic computer-generated randomization without stratification, the patients were randomized into 2 groups, distal (n = 30) and proximal group (n = 30) (Figure 1). While the interventions were performed openly by the treating physicians, the outcome assessors who conducted follow-up evaluations were blinded to group allocation.

### 2.2. Interventions

All procedures were performed by the same two physicians in the operating room, under sterile conditions, and with monitoring during the procedure. Patients were placed in a prone position with the head and neck slightly flexed and the application area was sterilized. A TOSHIBA Aplio 500 Ultrasound (US) device and 22-gauge, 5 cm RF cannulas with a 5 mm active tip were used in both groups. For proximal approach, the GON was targeted at the C2 level, between the inferior obliquus capitis and semispinalis capitis muscles. For distal approach, the GON was identified at the superior nuchal line near the occipital artery. After visualizing the electrode tip placed close to target, a sensory stimulation test was performed using an RF generator (NeuroTherm NT1100). In both groups, PRF was applied bilaterally at 5 Hz and 5 ms for 360 s at 45 V, ensuring electrode tip temperatures did not exceed 42 ºC.

### 2.3. Variables and endpoints

Participants’ headache diaries were assessed to record headache related outcomes: duration (hours per episode), severity (VAS score, 0–10), frequency (days/month), and the total number of headache days per month. All parameters were reassessed at 1-, 2-, and 3-months posttreatment to monitor the treatment’s impact over time. The primary endpoint was changes in headache frequency evaluated at baseline and each follow-up (1-, 2-, and 3-months posttreatment). Secondary endpoints included duration, severity, total number of headache days, frequency of severe (VAS ≥ 5) and mild (VAS < 5) attacks, and any side effects.

### 2.4. Power analysis

In this study, sample size estimation was performed using G*power version 3.1 (Heinrich-Heine-Universität Düsseldorf, Düsseldorf, Germany). We assumed a baseline mean headache frequency of 12 days per month with a standard deviation (SD) of 5 days. Assuming a 30% difference in headache frequency postintervention, we estimated that 25 patients per group would be required to detect a significant difference, maintaining a statistical power of β = 0.80 and a significance level of α = 0.05.

### 2.5. Statistical analysis

Data analysis was performed using Jamovi Project (2022, Jamovi Version 2.3, Computer Software). Descriptive statistics were used to summarize the demographic and clinical characteristics of the participants. Continuous variables were reported as means and standard deviations (SD) or medians and interquartile ranges (IQR) as appropriate. Categorical variables were summarized as frequencies and percentages.

Between-group comparisons were performed cross-sectionally at each time point (baseline, 1st, 2nd, and 3rd months) using the Mann-Whitney U test. Within-group differences were assessed longitudinally over time within each group using the Friedman test. We also assessed changes in headache duration, severity, frequency, and the number of headache days across the two study groups. Delta values, calculated as the change from baseline to each follow-up time point (1st, 2nd, and 3rd months), were compared between groups at each follow-up using the Mann-Whitney U test. To quantify the magnitude of between-group differences of key variables, the effect size was calculated using the formula r = Z / √N, where *Z* is the standardized test statistic obtained from the Mann–Whitney U test, and *N* is the total number of observations across both groups. Effect sizes were interpreted based on Cohen’s guidelines, where *r* = 0.1 indicates a small, *r* = 0.3 a medium, and *r* = 0.5 a large effect. A p-value of < 0.05 was considered statistically significant. All analyses were conducted on an intention-to-treat basis.

## 3. Results

From March 2023 to March 2024, 71 patients were screened for eligibility, 8 were excluded and 3 patients did not consent to participate. A total of 60 patients with episodic or chronic migraine, 30 in each distal and proximal group, were evaluated in the study (Figure 1). No significant differences were observed between groups in terms of age or sex. BMI and migraine duration was comparable between groups. The proximal group, however, reported a significantly higher median monthly attack frequency (15.0 vs. 9.5, p = 0.007), severe attacks frequency (8.5 vs. 4.5, p < 0.001), and more headache days per month (20 vs. 15, p =0.019). No significant differences were observed in medication use for prophylaxis, daily life impacts, absenteeism days per month, or sleep disturbances (p > 0.05) (Table 1).

Over time, both groups showed significant reductions in headache duration, severity, frequency, and the number of headache days (Friedman test, p < 0.001) (Table 2).

**Figure 2:**
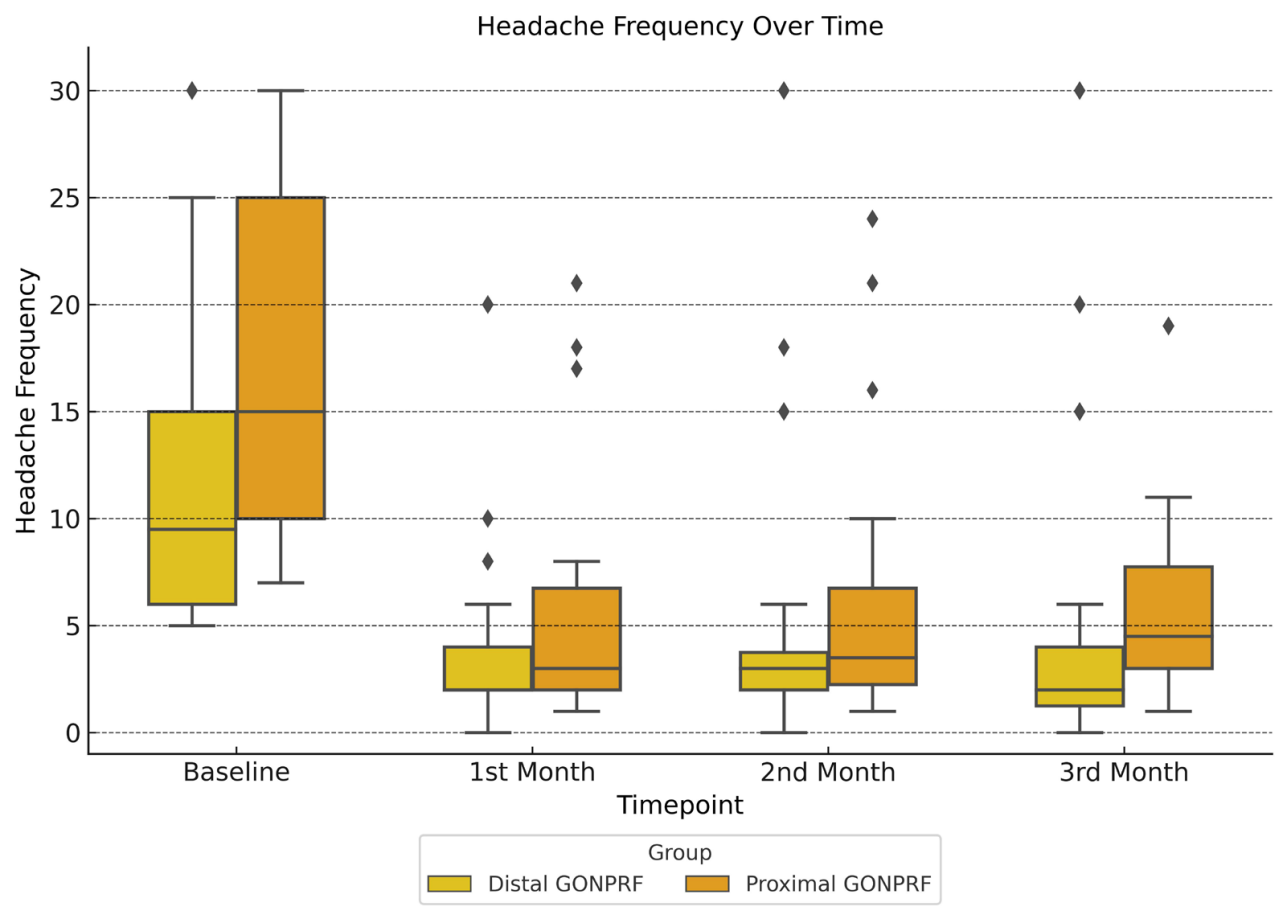
Monthly changes in headache frequency between distal and proximal GON PRF groups over 3 months.

At baseline, the proximal group had a higher attack frequency. By the first month, both groups experienced reductions in headache frequency, with medians of 2 attacks for the distal group and 3 attacks for the proximal group (p = 0. 163). In the second month, the distal group maintained a lower frequency (3 attacks vs. 3.5 attacks, p = 0.036), and by the third month, this difference was statistically significant (2 attacks vs. 4.5 attacks, p = 0.034) (Figure 2).

Initially, there was no significant difference in headache duration between the groups (median 24 h, p = 0.450). By the first month, the distal group experienced shorter headache durations compared to the proximal group (7 h vs. 12 h, p = 0.036). This trend persisted in the second and third months, with the distal group continuing to report shorter durations (p = 0.011 and p = 0.001, respectively).

**Figure 3:**
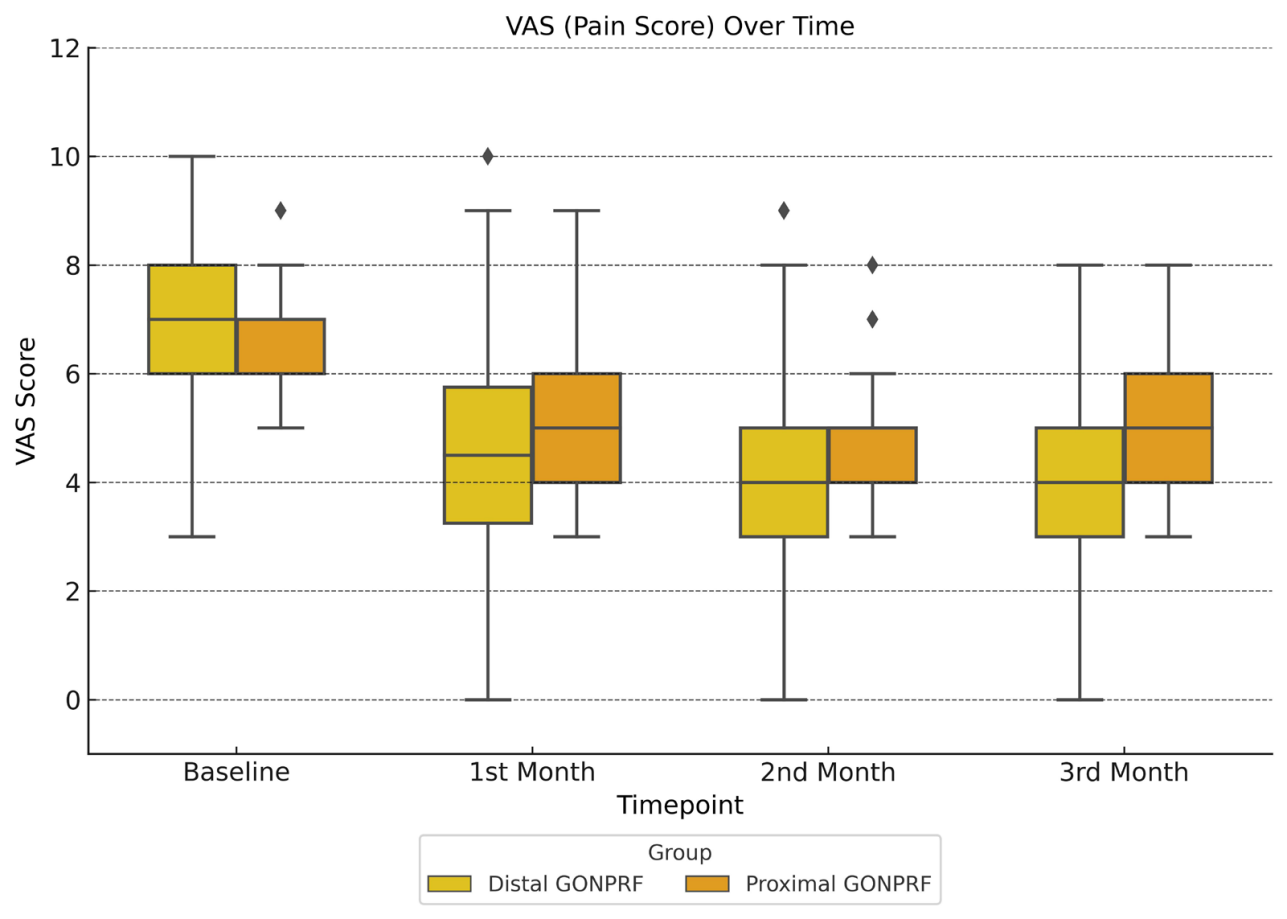
Monthly changes in visual analog scale (VAS) scores between distal and proximal GON PRF groups over 3 months.

At baseline, the mean VAS scores were similar between the groups. By the first month, both groups showed a reduction in VAS scores, but the difference between them was not statistically significant (p = 0.320). By the second and third months, however, the distal group had significantly lower mean VAS scores (4.13 and 4.03) compared to the proximal group (4.93 and 5.10, p = 0.030 and p = 0.020, respectively) (Figure 3).

Regarding the number of headache days per month, the proximal group initially had a higher median (20 days vs. 15 days, p = 0.019). By the first month, both groups showed significant decreases, with medians of 2.5 days for the distal group and 3.0 days for the proximal group (p = 0.281). In the second and third months, the distal group continued to show fewer headache days (3 days each month) compared to the proximal group (3.5 and 4.5 days, p = 0.067 and p = 0.060, respectively) (Table 2).

Delta analysis revealed that median changes of headache duration and frequency were not statistically significant between the groups at any time point (p > 0.05). However, there was a trend toward significance in headache frequency reduction during the first month, with the proximal group showing a median reduction of 11.0 attacks compared to 7.0 attacks for the distal group (p = 0.055) (Table 3, Figure 2)

In terms of VAS score reductions, delta analysis (Table 3) showed that the 3rd month change favored the Distal group (r = 0.325, p = 0.011), demonstrating a medium effect size. In the first month, reductions were similar (p = 0.431), and in the second month, there was a trend toward significance favoring the distal group (p = 0.055). (Table 3, Figure 3)

Regarding the number of headache days, the proximal group showed consistently larger reductions across all time points. In the first month, the proximal group experienced a median reduction of 14.5 days compared to 10.5 days in the distal group (r=0.267, p = 0.038), reflecting a low-to-medium effect size. This trend continued, with a trend towards significance in the second month (p = 0.055) and a statistically significant difference by the third month (14.0 vs. 9.5 days, p = 0.039).

For severe headaches, significant differences were observed between the groups across all time points. Delta analysis indicated that the proximal group had a more substantial reduction in the frequency of severe headache attacks compared to the distal group at all intervals. At the 1st month, the reduction in severe attack frequency was significantly greater in the proximal GON PRF group compared to the distal group (r = 0.368, p = 0.004), indicating a medium effect size. Similarly, at the 2nd month, this difference remained significant (r = 0.330, p = 0.010), also representing a medium effect. At the 3rd month, this difference remained significant with a low to medium effect size (r = 0.294, p = 0.022). In contrast, no statistically significant differences in reductions were observed between the groups for the frequency of mild headaches or absenteeism over the study period.

In the distal group, adverse effects were observed in a total of four patients throughout the study period. Specifically, dizziness was reported by three patients, while nausea occurred in one patient. In the proximal group, adverse events were documented in four patients as well. Among these, one patient experienced pruritus, two reported dizziness, and one reported nausea. Overall, the incidence of side effects was similar between the two groups, with dizziness being the most reported symptom.

## 4. Discussion

This randomized controlled trial compared the efficacy and safety of GON PRF treatment with US-guided proximal and distal approaches in patients with refractory episodic or chronic migraine. We demonstrated that both the proximal and distal GON PRF approaches showed significant reductions in headache frequency, severity, and duration, as well as the total number of headache days over a 3-month period. Notably, while both techniques were effective, they exhibited distinct efficacy profiles with subtle nuances in their performance. Both methods had acceptable safety profiles.

Due to a functional connection between the sensory occipital segments and the trigeminal nociceptive system, GON block is recognized as an effective treatment option for reducing headache severity and frequency in migraine patients who either do not respond to medical therapy or cannot tolerate prophylactic treatment due to side effects [7,10,11]. The procedure can be performed using either a proximal approach, targeting the suboccipital region between the semispinalis capitis and inferior obliquus capitis muscles,, or a distal approach, focusing on the superior nuchal line. When Greher et al. first introduced the proximal technique, they argued that it could offer greater precision compared to the distal approach [15]. However, the comparative efficacy of these two methods remains debated, as studies have reported mixed results [16–18]. For instance, Kissoon et al. reported that the proximal GON block provided superior pain relief compared to the distal approach. Notably, the proximal technique in their study was performed under ultrasound guidance, while the distal block relied on anatomical landmarks [17]. Ultrasound guidance is known to enhance procedural accuracy and reduce the risk of vascular puncture, as demonstrated in the randomized placebo-controlled trial by Palamar et al., which found US-guided distal GON block effective in treating refractory migraine in a monthly follow-up study and recommended it to improve procedural precision [19]. In our study, both treatment approaches were performed under ultrasound guidance to ensure accuracy.

Several studies have demonstrated that GON PRF is both effective and safe for migraine management [20–25]. A randomized controlled trial involving 67 patients (including 25 migraine sufferers) compared the efficacy of GON PRF with GON block (local anesthetic + steroid) showed a significant reduction in pain for up to six months following PRF treatment, though there were no significant differences in secondary outcomes like headache impact or sleep quality [20]. Unlike our study, the trial used the anatomical landmark technique for distal PRF applications, while we employed ultrasound and Doppler imaging, focusing on three-month outcomes. Furthermore, a randomized controlled trial comparing single-dose GON block with a combination of GON block and PRF reported that the combined treatment was significantly more effective over six months [21]. In this trial, similar to our study, the distal approach was US-guided. A retrospective cohort study examined 27 migraine patients who had insufficient responses to GON block and were treated with distal GON PRF. The results showed significant improvements in monthly headache frequency, attack duration, and total analgesic consumption up to six months posttreatment compared to baseline [22]. The literature contains four studies addressing the proximal application of GON PRF for migraine treatment [23–26]. In a case report by Kwak et al., two chronic migraine patients who were resistant to GON block and botulinum toxin injections showed reduced headache intensity for up to three months, although headache duration and frequency remained largely unchanged [23]. In the first retrospective cohort study on this topic, proximal PRF was administered to 25 chronic migraine patients [24] leading to significant improvements in headache severity, frequency, and duration for up to three months. A small case series (n = 6) investigated the combined effects of ultrasound-guided PRF and onabotulinumtoxin A (BTA) injections [25]. The study reported significant reductions in headache intensity and frequency, sustained for up to six months.

Consistent with our findings, a recent study comparing proximal and distal GON PRF treatments confirmed that both ultrasound-guided approaches are effective and safe for migraine treatment [26]. However, unlike our study, this research also evaluated procedure duration and procedural pain levels. The authors reported that the distal approach offered significant advantages regarding patient comfort (due to lower procedural pain) and clinical applicability (due to shorter procedure time), recommending it for consideration in clinical decision-making.

Our study clearly demonstrated that both approaches provided distinct and clinically significant benefits on their own over the 3-month follow-up period. Statistically highly significant reductions compared to baseline were observed in both groups for headache-related outcomes. These findings support that both US-guided GON PRF techniques possess a substantial effect in reducing migraine burden. However, the direct comparison of the methods’ efficacy over time revealed some statistically significant differences, detailed below, which possessed low to medium effect sizes. It is these nuanced differences that raise the potential for tailored management. The distal group showed greater reductions in VAS scores by the later months of the study, while the proximal group exhibited consistently larger decreases in the number of headache days at the first month and the frequency of severe headache attacks across all time points. No statistically significant differences were noted between the groups in reductions for mild headache frequency or absenteeism. Baseline assessments revealed that participants in the proximal group had a higher disease burden, characterized by significantly greater monthly migraine attack frequency, a higher number of severe attacks, and more headache days compared to the distal group. Thus, the proximal approach appears to be particularly suitable for patients with a higher migraine burden and could be considered as a first-line option for this subgroup. The greater reductions in VAS scores observed in the distal group by the later months suggest that this approach may be particularly effective in alleviating pain intensity over time.

Both methods demonstrated a favorable safety profile. Adverse effects were minimal and comparable between groups, with the most reported side effect being dizziness, affecting three patients in the distal group and two in the proximal group. Other minor adverse effects included nausea (one patient in each group) and pruritus (one patient in the proximal group). The low incidence of side effects supports the safety and tolerability of both proximal and distal GON PRF approaches, with no significant difference in adverse event profiles.

This study has several limitations that should be considered when interpreting the results. First, the sample size, although sufficient to detect within-group changes, may limit the generalizability of our findings to broader populations. Second, despite randomization, a significant imbalance in baseline migraine burden was observed between the groups, with the proximal group reporting significantly higher median monthly attack frequency, severe attack frequency, and total headache days. This baseline difference represents a potential confounder, which could influence the comparison of treatment effects. Third, the follow-up period was limited to three months, which may not fully capture the long-term efficacy and sustainability of treatments. Fourth, while both groups showed clinically meaningful improvements, the lack of a sham or placebo control group means that some of the observed effects could be attributed to the placebo effect or patient expectation. Future studies with larger sample sizes addressing potential baseline imbalances through stratified randomization, longer follow-up periods, and the inclusion of appropriate control groups are warranted to further validate these findings.

## 5. Conclusions

In conclusion, this study demonstrates that both distal and proximal GON PRF treatments are effective in reducing migraine frequency and severity in patients with chronic or episodic migraine. While both approaches yielded significant improvements, proximal GON PRF was particularly effective in reducing the frequency of severe attacks and overall headache days, making it a promising option for patients with a higher baseline disease burden. Overall, our findings underscore the potential for GON PRF interventions as safe, minimally invasive options in migraine management, and highlight the value of tailoring treatment strategies to individual patient profiles. Further research with larger cohorts and longer-term follow-up is needed to refine and expand upon these promising results.

## Figures and Tables

**Table 1:** Comparison of demographic and clinical characteristics between distal GON PRF and proximal GON PRF groups.

Characteristic	Distal GON PRF	Proximal GON PRF	p-value
**Age, years (mean ± SD)**	42.4 (10.5)	40.7 (10.3)	0.546
**Female, n (%)**	27 (90.0%)	23 (76.7%)	0.166
**BMI, kg/m** **^2^** ** (mean ± SD)**	25.0 (3.7)	26.2 (4.5)	0.260
**Educational status, n (%)**			0.161
Elementary	9 (30.0%)	7 (23.3%)	
Middle School	4 (13.3%)	0 (0.0%)	
High School	5 (16.7%)	7 (23.3%)	
University	12 (40.0%)	16 (53.3%)	
**Headache side localization, n (%)**			**0.019**
Right	4 (13.3%)	2 (6.7%)	
Left	0 (0.0%)	1 (3.3%)	
Alternate	8 (26.7%)	19 (63.3%)	
Bilateral	18 (60.0%)	8 (26.7%)	
**Migraine duration, years**	15.0 (12.75)	16.5 (18.5)	0.227
**Headache duration per episode, hours**	24.0 (9.0)	24.0 (24.0)	0.441
**Attack frequency per month**	9.5 (9.0)	15.0 (15.0)	**0.007**
**Headache days per month**	15.0 (10.0)	20.0 (15.0)	**0.019**
**Average Headache Severity (VAS)**	7.0 (1.75)	7.0 (1.0)	0.310
**Maximum Headache Severity (VAS)**	9 (1.0)	9 (1.0)	0.070
**Severe attack frequency per month**	4.5 (4.75)	8.5 (3.0)	**<0.001**
**Mild attack frequency per month**	5.0 (5.0)	7.0 (9.0)	0.563
**Emergency department visits/month**	1.0 (2.0)	2.0 (3.0)	0.081
**Daily life impact**			
Absenteeism, days/month	3.5 (3.0)	3.0 (3.0)	0.834
Sleep disturbance, n (%)	21 (70.0%)	23 (76.7%)	0.770
Daytime sleepiness days per month	3.0 (4.0)	4.0 (4.5)	0.167

Values are presented as mean (SD) for Age and BMI, median (IQR) for other continuous variables, or number (n, %) for categorical variables.

Statistically significant difference (p < 0.05) between the distal GON PRF and proximal GON PRF groups is indicated in bold.

Abbreviations: BMI, Body mass index; GON PRF, Greater occipital nerve pulsed radiofrequency; IQR, Interquartile range; n, Number; SD, Standard deviation; VAS, Visual analog scale.

**Table 2:** Comparison of headache-related measures (duration, VAS, frequency, and days) between groups at time points.

Measure/Time Point	Distal Mean (SD)	Distal Median (IQR)	Proximal Mean (SD)	Proximal Median (IQR)	p-value
**Headache duration per episode, hours**					
Baseline	27.7 (17.9)	24.0 (9)	31.0 (17.7)	24.0 (24)	0.450
1st month	10.0 (8.1)	7.0 (10)	14.0 (8.4)	12.0 (16)	**0.036**
2nd month	8.8 (9.6)	6.0 (7)	13.3 (8.6)	10.0 (15.5)	**0.011**
3rd month	7.8 (9.5)	5.0 (4)	14.0 (8.7)	12.0 (16)	**0.001**
**Average headache severity (VAS)**					
Baseline	7.33 (1.73)	7.00 (1.75)	6.97 (0.93)	7.0 (1)	0.310
1st month	4.87 (2.70)	4.5 (2.5)	5.10 (1.30)	5.0 (2)	0.320
2nd month	4.13 (2.50)	4.0 (2)	4.93 (1.14)	5.0 (1)	**0.030**
3rd month	4.03 (2.25)	4.0 (2)	5.10 (1.35)	5.0 (2)	**0.020**
**Attack frequency per month**					
Baseline	12.23 (8.12)	9.5 (9)	17.40 (8.36)	15.0 (15)	**0.007**
1st month	3.97 (4.88)	2.0 (2)	5.17 (5.12)	3.0 (4.75)	0.163
2nd month	4.30 (6.20)	3.0 (1.75)	5.87 (5.59)	3.5 (4.5)	**0.036**
3rd month	4.73 (6.68)	2.0 (2.75)	5.97 (5.16)	4.5 (4.75)	**0.034**
**Headache days per month**					
Baseline	16.03 (8.41)	15.0 (10)	20.93 (6.98)	20.0 (15)	**0.019**
1st month	4.27 (4.97)	2.5 (2)	5.30 (5.22)	3.0 (4.75)	0.281
2nd month	4.77 (6.64)	3.0 (2)	6.00 (5.74)	3.5 (4.5)	0.067
3rd month	5.47 (7.83)	3.0 (2.75)	6.17 (5.43)	4.5 (4.75)	0.060

Values are presented as mean (SD) and median (IQR).

Statistically significant difference (p < 0.05) between the distal and proximal groups at that specific time point is indicated in bold.

Note: All within-group comparisons over time (Baseline vs. 1st, 2nd, 3rd Month) were statistically significant for both groups (Friedman test, p < 0.001).

Abbreviations: IQR, Interquartile range; SD, Standard deviation; VAS, Visual analog scale.

**Table 3:** Changes in headache-related measures (duration, VAS, frequency, and days) overtime for groups.

Time Point	Measure	Change in Distal GON PRF Median (IQR)	Change in Proximal GON PRF Median (IQR)	p-value
	**Headache duration per episode, hours**			
1st month		−18.0 (19.0)	−12.0 (33.0)	0.490
2nd month		−17.0 (16.0)	−12.00 (33.0)	0.380
3rd month		−18.5 (16.5)	−12.0 (33.3)	0.350
	**Average headache severity (VAS)**			
1st month		−2.0 (3.5)	−2.0 (1.0)	0.431
2nd month		−3.0 (3.5)	−2.0 (0.75)	0.055
3rd month		−3.0 (2.75)	−2.0 (1.75)	**0.011**
	**Attack Frequency per month**			
1st month		−7.0 (6.0)	−11.0 (12.5)	0.055
2nd month		−7.0 (6.0)	−11.0 (12.5)	0.110
3rd month		−6.50 (6.0)	−10.5 (11.5)	0.100
	**Headache days per month**			
1st month		−10.5 (10.0)	−14.5 (8.5)	**0.038**
2nd month		−10.0 (8.5)	−14.5 (8.5)	0.055
3rd month		−9.5 (10.25)	−14.0 (8.0)	**0.039**
	**Severe attack frequency per month**			
1st month		−3.0 (4.75)	−6.0 (3)	**0.004**
2nd month		−3.5 (3.5)	−6.5 (4.75)	**0.010**
3rd month		−4.0 (4.5)	−6.0 (4)	**0.022**
	**Mild attack frequency per month**			
1st month		−4.0 (5.75)	−4.0 (10.5)	0.716
2nd month		−3.0 (6.75)	−3.5 (10.0)	0.678
3rd month		−2.0 (6.75)	−4.0 (10.0)	0.583
	**Absenteeism days/month**			
1st month		−2.0 (4)	−3.0 (2.75)	0.215
2nd month		−3.0 (3)	−3.0 (2.75)	0.596
3rd month		−3.0 (3)	−3.0 (2.75)	0.367

Values represent the change from baseline presented as median (IQR).

A statistically significant difference (p < 0.05) in the change between the distal and proximal groups is indicated in bold.

Abbreviations: IQR, Interquartile range; VAS, Visual analog scale.
